# Tribbles pseudokinase 3 promoted renal fibrosis by regulating the expression of DNA damage-inducible transcript 3 in diabetic nephropathy

**DOI:** 10.17305/bb.2024.10419

**Published:** 2024-12-01

**Authors:** Lulu Kong, Liusha Kong, Peipei Li, Li Gao, Hongqin Ma, Bimin Shi

**Affiliations:** 1 Department of Endocrinology, The First Affiliated Hospital of Soochow University, Suzhou, China; 2 Department of Endocrinology, The Affiliated Hospital of Xuzhou Medical University, Xuzhou, China; 3 Department of Nephrology, The Affiliated Hospital of Xuzhou Medical University, Xuzhou, China; 4 Department of General Surgery, The Affiliated Hospital of Xuzhou Medical University, Xuzhou, China

**Keywords:** Diabetic nephropathy (DN), tribbles pseudokinase 3 (TRIB3), DNA damage-inducible transcript 3 (DDIT3), inflammatory response, extracellular matrix (ECM) accumulation

## Abstract

Diabetic nephropathy (DN) is a severe complication of prolonged diabetes, impacting millions worldwide with an increasing incidence. This study investigates the role of tribbles pseudokinase 3 (TRIB3), a protein implicated in the progression of DN, focusing on its mechanisms underlying glomerular damage. Through analysis of the Gene Expression Omnibus (GEO) database, we identified TRIB, among differentially expressed genes (DEGs) in streptozotocin (STZ)-treated C57BL/6J mice. Both in vitro and in vivo experiments were conducted to examine the effects of TRIB3 inhibition on high glucose (HG)-induced damage in podocytes and DN mouse models. The results demonstrated that TRIB3 inhibition reduced inflammatory responses and extracellular matrix (ECM) production in MPC5 cells, mediated by the downregulation of DNA damage-inducible transcript 3 (DDIT3) - a critical regulator of proinflammatory cytokine secretion and ECM synthesis. Inhibiting TRIB3 decreased inflammatory factors and ECM deposition in diabetic mice in vivo, confirming its pivotal role in DN pathogenesis. These findings indicate that TRIB3 and its interaction with DDIT3 contribute significantly to DN by promoting inflammatory cascades and ECM accumulation, presenting potential therapeutic targets for managing the disease.

## Introduction

Diabetic nephropathy (DN) is a common and serious complication of diabetes mellitus (DM), particularly in individuals with poorly controlled blood sugar levels over an extended period, which has a widespread impact on millions of individuals globally [1, 2]. While efforts to prevent and slow the advancement of DN by rigorously managing blood glucose and blood pressure have been undertaken, the outcomes of these interventions remain unsatisfactory [3–5]. DN is characterized by notable hallmarks such as thickening of the glomerular basement membrane, accumulation of matrix in both the mesangium and tubule interstitium, and dysfunction or reduction of podocytes. These feature changes contribute to the progression of renal fibrosis, even end-stage renal disease [6, 7]. As the incidence and prevalence of this debilitating condition continue to surge, the urgency to comprehend its underlying molecular intricacies becomes ever more critical.

Several studies have highlighted tribbles pseudokinase 3 (TRIB3), a member of the tribbles family of proteins, characterized by their unique pseudokinase domain [8–10]. Unlike traditional kinases, TRIB3 lacks the canonical adenosine triphosphate (ATP)-binding pocket and catalytic residues, leading to its classification as a pseudokinase. Instead of direct catalytic activity, TRIB3 functions as a molecular scaffold, orchestrating intricate interactions with various transcriptional mediators and signaling molecules [11–13]. The dysregulation of TRIB3 gene expression has been linked to several diseases, underscoring its importance in disease pathogenesis. For example, altered TRIB3 expression has been associated with diabetes, hepatitis, neurodegenerative disorders, and tissue fibrosis [9, 11, 14, 15]. Investigative studies have unveiled that thwarting the expression of TRIB3 can yield a reduction in glomerular damage and the presence of albuminuria in experimental diabetic animal models [16, 17]. Increasing evidence suggests that TRIB3 regulated protein kinase B (AKT) and/or mitogen-activated protein kinase (MAPK) pathways in glucose homeostasis, mediating insulin resistance, and regulating cellular stress responses under diabetic conditions [18–20]. However, the precise mechanisms through which TRIB3 orchestrates these transformative effects remain unclear.

DNA damage-inducible transcript 3 (DDIT3), a member of the C/EBP family, is a transcription factor with diverse biological functions, particularly in response to cellular stress [21, 22]. It plays a crucial role in regulating cellular adaptation and survival during stressful conditions. DDIT3 is a key player in the unfolded protein response (UPR), a cellular pathway activated in response to endoplasmic reticulum (ER) stress, which is a hallmark of DN [23, 24].

In our study, we analyzed the transcriptional profiles of podocytes from mice with or without streptozotocin (STZ), using data from the Gene Expression Omnibus (GEO) database. TRIB3 was identified as a crucial stress-responsive pseudokinase upregulated in podocytes from diabetic mice. In vitro and in vivo experiments were performed to investigate the roles and underlying mechanisms of TRIB3 in high-glucose (HG) induced podocytes, which revealed a critical role for the TRIB3/DDIT3 axis in the development and progression of DN.

## Materials and methods

### Bioinformatics analysis

The three gene expression profiles (GSE173989, GSE197699, and GSE60038) were acquired from the GEO (https://www.ncbi.nlm.nih.gov/geo/) for the differentially expressed genes (DEGs) in podocyte translatome from mice with or without STZ-induced diabetes [25–27]. DEGs were analyzed using easyGEO (https://tau.cmmt.ubc.ca/eVITTA/easyGEO/#) with genes with fold change (FC) > 1 and *P* value < 0.05 considered to have significant differences of expression [28].

GEO datasheet (GSE212489 and GSE107094) was used to analyze the DEGs after TRIB3 knockdown.

### Animal experiments

Eight-week-old male C57BL/6 mice (32–36 g) were purchased from the Shanghai Slack Laboratory Animal Co., Ltd. (Shanghai, China) and maintained under a 12-h light-dark cycle with free access to tap water and commercial chow. Sixty-four mice were randomly assigned into four groups: control group (*n* ═ 8, injected with saline), model group (*n* ═ 16, injected with STZ), SCR group (*n* ═ 16, model group injected with negative control lentivirus), and shTRIB3 group (*n* ═ 16, model group injected with the TRIB3-shRNA lentivirus). Mice were rendered diabetic by 100 mg/kg STZ (Sigma-Aldrich, USA) intraperitoneal injection as previously reported [29]. DM was confirmed by levels of blood glucose and periodic acid-Schiff (PAS) staining. The negative control lentivirus or TRIB3-shRNA lentivirus was injected into the tail vein with 3 × 10^8^ IFU/mouse. Body weight was monitored weekly for 12 weeks. The mice were euthanized at 12 weeks with an intraperitoneal injection of sodium pentobarbital (50 mg/kg body weight). Blood samples were harvested for biochemical parameters and kidneys were collected for histological examination and immunohistology assays. All animal procedures in this study were approved by the Animal Care and Use Committee of the Department of Laboratory Animal, Shanghai ZY (Permit No. SHZY-20221016D). All animal experiments complied with the ARRIVE guidelines and were performed in accordance with the U.K. Animals (Scientific Procedures) Act, 1986, and associated guidelines, EU Directive 2010/63/EU for animal experiments.

### Plasmid construction and transduce

The short hairpin RNAs (shRNAs) targeting TRIB3 were constructed using pLKO.1-TRC cloning vector. The hairpin structure shRNA oligonucleotide was designed as follows: forward oligo 5′-CCGGAAGGAAGAAACCGTTGGAGTTCTCGAGGTATAGCTTAACGTAGGCATTTTTTTG-3′, and reverse oligo 5′-AATTCAAAAAAATGCCTACGTTAAGCTATACCTCGAGAACTCCAACGGTTTCTTCC-3′. A non-sense scramble oligonucleotide (SCR) shRNA was used as the negative control. Lentiviral particles were produced by transfected SCR or TRIB3-shRNA together with two packaging vectors, psPAX2 and pMD2.G into 293T cells. After filtering with a 0.45 µm pore size membrane filter, the solution was added to each well and selected with 2 mg/L puromycin.

To overexpress DDIT3, the full-length of DDIT3 was amplified and cloned into pcDNA3.1 (Invitrogen, Carlsbad, CA, USA). MPC5 cells with TRIB3 silencing were transfected with pcDNA3.1-DDIT3 or the empty control (control) via Lipofectamine 3000 (Invitrogen) according to the manufacturer for cell transfections.

### Cell culture

Mouse-immortalized podocyte MPC5 cells were obtained from the Cell Bank of Chinese Academy of Sciences (Shanghai, China) and cultured as described previously [30]. For in vitro assay, MPC5 cells were treated with 5.5 mM D-glucose (Normal), 30 mM D-glucose (HG), and a combination of HG and SCR or shTrib3. After one day of transfection, the cells were stimulated with HG (final concentration 30 mmol/L glucose) for 48 h and then harvested. To adjust the glucose concentration from 5.5 to 30 mM in a cell culture medium, approximately 11.35 mL Gibco™ Glucose Solution (200 g/L) to 5.5 mM medium was added.

### Cell viability assay

The viability of MPC5 cells was determined using a Cell Counting Kit-8 (CCK8) solution (Dojindo, Tokyo, Japan) according to the manufacturer’s protocol. In brief, approximately 1 × 10^4^ cells were seeded into 96-well plates and 10-µL CCK8 solution was added to each well and incubated for another 2 h at 37 ^∘^C. Then the optical density value was measured at a wavelength of 450 nm using a microplate reader (Bio-Rad, CA, USA).

### Flow cytometry

Cell apoptosis was measured using FITC Annexin V Apoptosis Detection Kit I (BD Pharmingen, NJ, USA). Cells were harvested and stained with Annexin-V-FITC/PI solution, then measured by a FACSCalibur flow cytometer (BD).

### Tunnel assay

One-step TUNEL In Situ Apoptosis Kit (Elabscience Biotechnology Inc., Hubei, China) was used to detect the apoptotic level of kidney tissue according to the manufacturer’s instructions. In brief, the kidney tissue sections from each group were treated with protease K working fluid to restore the tissue. TdT was added after the membrane was broken and mixed with dUTP. The nucleus was stained with DAPI and the slides were fixed with an anti-fluorescence quenching agent after incubation at room temperature. Images of the apoptotic cells were visualized by using a laser confocal microscope (TCS SP8 STED; Leica, Wetzlar, Germany).

### RNA extraction and reverse transcription-quantitative polymerase chain reaction (RT-qPCR) analysis

Total RNA was extracted using TRIzol reagent (Invitrogen) and reverse transcribed into cDNA using the PrimeScript reverse transcriptase reagent kit (Takara, Kusatsu, Japan). Real-time PCR was performed using Fast SYBR™ Green Master Mix (Invitrogen) with the following primers on the 7900HT Fast Real-Time PCR System (Applied Biosystems, CA, USA). The primers for TRIB3 were: forward primer 5′-GGCTCTCGGCTCCTTTACATC-3′, reverse primer 5′-CCTCGGACTCTGGGATACCG-3′. For collagen IV, forward primer 5′-TCCGGGAGAGATTGGTTTCC-3′, reverse primer 5′-CTGGCCTATAAGCCCTGGT-3′. For fibronectin, forward primer 5′-ATGGCGACGGTATTCTGTAAAG-3′, reverse primer 5′-TTGGCAGTTGGTCAATCACAT-3′. For laminin, forward primer 5′-CCCCGTCCTTGGATGTACCT-3′, reverse primer 5′-CAGTAGGGTTGAGGGCTATGC-3′. For glyceraldehyde 3-phosphate dehydrogenase (GAPDH) forward primer 5′-TGACCTCAACTACATGGTCTACA-3′, reverse primer 5′-CTTCCCATTCTCGGCCTTG-3′. As the endogenous control, GAPDH was used.

### Enzyme-linked immunosorbent assay (ELISA)

Commercial ELISA kits including Mouse TNF-α ELISA Kit (Catalog: PT512, Beyotime Biotechnology, Zhejiang, China), Mouse IL-6 ELISA Kit (Catalog: PI326, Beyotime Biotechnology), and Mouse IL-1β ELISA Kit (Catalog: PI301, Beyotime Biotechnology) were used to determine the concentrations of tumor necrosis factor-alpha (TNF-α), interleukin-6 (IL-6), and interleukin-1 beta (IL-1β) in the cell supernatant and serum according to the manufacturer’s instructions.

### Renal biochemical assessments

Nocturnal urine and serum were collected as previously described [31]. The content of creatinine, blood urea nitrogen (BUN), and glycated serum protein (GSP) in serum and 24 h urine protein (UP) were measured by a 7100 automatic biochemical analyzer (Hitachi, Japan).

### Histopathological analysis and immunohistochemical staining

The kidney tissues mentioned above were stained with hematoxylin and eosin (H&E) and PAS for routine morphological observation following standard staining protocols. For immunohistochemical (IHC) staining, slices were dewaxed, treated with citrate buffer (pH 6.0), and incubated overnight at 4 ^∘^C with TRIB3 and DDIT3 primary antibodies. After a horseradish peroxidase-conjugated secondary antibody treatment, sections were stained with 3,3′-diaminobenzidine (DAB) and hematoxylin. Images were obtained using a microscope (Invitrogen EVOS M7000) and processed with Image J software. Semiquantitative scoring was performed to assess renal tissue injury, with the renal injury score calculated by multiplying the intensity and area of the signal of five randomly taken images of renal cortex images.

### Western blot

Western blot analysis was performed using a standard protocol as previously described. In brief, total protein was extracted using RIPA buffer (Cell Signaling Technology, MA, USA) containing a protease inhibitor cocktail (Sigma-Aldrich, MO, USA). Adjusted protein amounts were separated on different concentrations of SDS-PAGE, and then transferred to PVDF membranes (Bio-Rad). After blocking in 5% dried skimmed milk, the membranes were incubated with the following antibodies at 4 ^∘^C overnight: anti-TRIB3 (Catalog: 13300-1-AP, Proteintech), Anti-DDIT3 (Catalog: 15204-1-AP, Proteintech), anti-collagen IV (Catalog: SAB4200500, Sigma-Aldrich), anti-fibronectin (Catalog: 66042-1-Ig, Proteintech), anti-laminin (Catalog: L9393, Sigma-Aldrich) and anti-GAPDH (Catalog: CW0100, Cwbio). GAPDH was used as an internal control. After treatment with the appropriate secondary antibody, the membranes were visualized with Immobilon Western Chemiluminescent HRP Substrate (Millipore, MA, USA). Protein density was calculated using ImageJ software (National Institutes of Health, MD, USA).

### Ethical statement

This study is based on an open-source database (TCGA and GEO), and approved by the Animal Care and Use Committee of the Department of Laboratory Animal, Shanghai ZY (Permit No. SHZY-20221016D).

### Statistical analysis

All data were expressed as the mean ± standard deviation (SD) from at least three independent measurements. GraphPad Prism 5 (GraphPad SoftwareInc., CA, USA) was used to assess the differences among groups using analysis of variance (ANOVA) followed by Newman–Keuls multiple comparison tests. *P* < 0.05 was considered statistically significant.

## Results

### TRIB3 was upregulated in HG-stimulated podocytes

**Figure 1.**
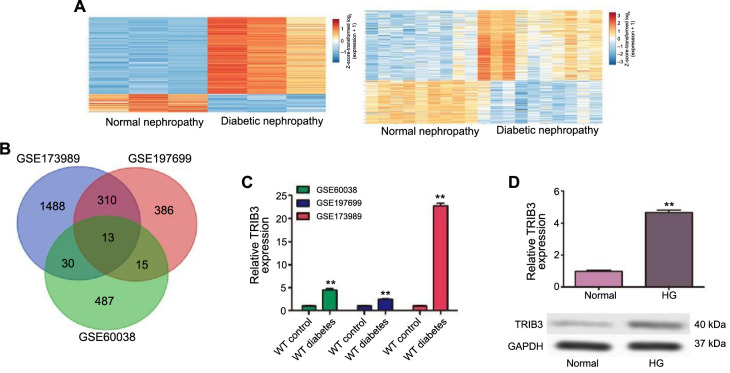
**TRIB3 expression in HG-induced MPC5 cells.** **P < 0.01. (A) Heat maps of the DEGs in podocytes, based on data from GSE197699 and GSE173989 datasheets; (B) Venn diagram illustrating DEGs identified from the GSE173989, GSE197699, and GSE60038 datasets to identify candidate genes in DN; (C) The mRNA level of TRIB3 based on GEO datasheets (GSE173989, GSE197699, and GSE60038); (D) qRT-PCR and western blot showing the expression of TRIB3 in MPC5 cells treated with 30-nM HG for 48 h. DEGs: Differentially expressed genes; DN: Diabetic neuropathy; TRIB3: Tribbles pseudokinase 3; HG: High glucose; qRT-PCR: Quantitative reverse transcription polymerase chain reaction; GEO: Gene Expression Omnibus; WT: Wild type; GAPDH: Glyceraldehyde 3-phosphate dehydrogenase.

To find the key genes involved in the progress of DN, three GEO datasets numbered GSE173989, GSE197699, and GSE60038 were selected for further analysis. As shown in Figure 1A, the heat map of the GSE197699 datasheet with >1-FC showed the DEGs in renal cortical tissues from wild-type (WT) mice and mice with STZ-induced diabetes. The heat map of the GSE173989 datasheet with >1-FC showed the DEGs in podocytes from podocytes isolated from WT mice and mice with STZ-induced diabetes. Thirteen overlapping DEGs (*TRIB3, KNG2, RCAN1, DDIAS, SYTL2, ATP2B4, PTGFRN, IRF1, NRP2, CTSS, TNC, RTP4,* and *THSD4*) were identified among the upregulated genes by Venn diagram (Figure 1B). Higher TRIB3 expression was observed in microarray datasets from GEO (GSE173989, GSE197699, and GSE60038) (Figure 1C). To further validate the expression level of TRIB3 in DB, we examined the transcriptional levels of TRIB3 via RT-qPCR and western blot in MPC5 cells with or without HG treatment. Consistent with the expression profile analysis with the database, we noticed a remarkable augmentation in the transcriptional level of TRIB3 in MPC5 cells with HG treatment (Figure 1D). These results demonstrated that TRIB3 levels were elevated under the HG state, thus suggesting that TRIB3 might play crucial regulatory roles in the progression of DN.

### TRIB3 was upregulated in DN

**Figure 2.**
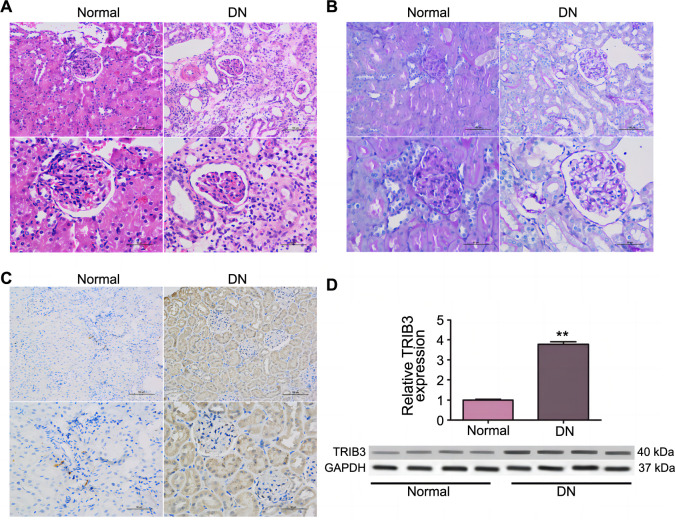
**TRIB3 upregulation in DN.** **P < 0.01. Representative images of normal and DN-affected mice (A) stained with H&E; (B) stained with PAS; (C) Immunohistochemical staining of TRIB3 in kidney sections; (D) qRT-PCR and western blot analysis of TRIB3 in mice kidney tissue. DN: Diabetic nephropathy; H&E: Hematoxylin and eosin; PAS: Periodic acid-Schiff; TRIB3: Tribbles pseudokinase 3; qRT-PCR: Quantitative reverse transcription polymerase chain reaction; GAPDH: Glyceraldehyde 3-phosphate dehydrogenase.

To confirm the upregulation of TRIB3 in the progression of DN, a chemically induced mouse model of DM was generated by intraperitoneal injection of STZ. After confirmation by blood glucose measurement (> 20 mmol/L), H&E and PAS staining revealed alterations in routine morphology in DN (Figure 2A and 2B). IHC staining showed that the level of TRIB3 was significantly upregulated in renal biopsy specimens of mice with DN (Figure 2C). Furthermore, qRT-PCR and western blot analysis revealed increased TRIB3 expression in the kidneys of STZ-induced diabetic mice (Figure 2D). All data indicated upregulation of TRIB3 might lead to renal tissue injury.

### TRIB3 silencing relieved HG-evoked podocyte apoptosis and proinflammatory cytokine expression

**Figure 3.**
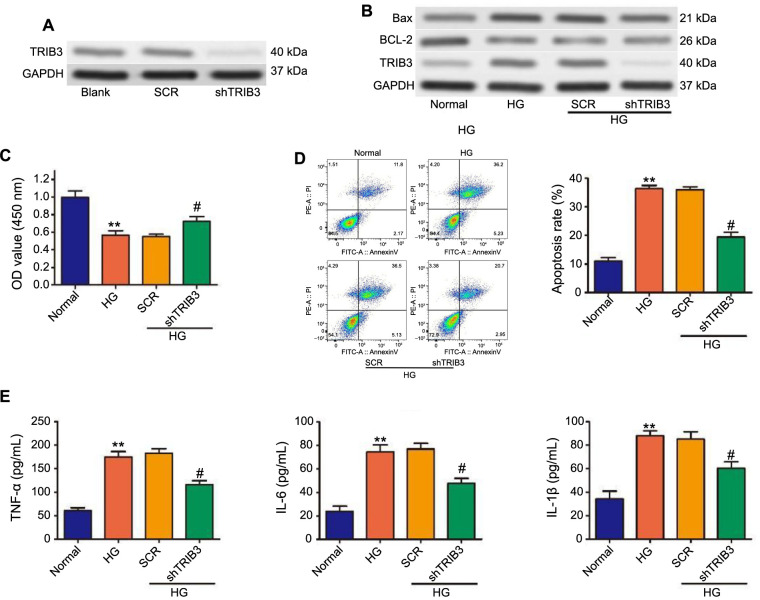
**Effects of TRIB3 knockdown on HG-evoked podocyte cell viability and inflammatory cytokine.** **P < 0.01 vs normal group; ^#^*P* < 0.01 vs the SCR group. (A) Expression of TRIB3 in MPC5 cells with or without TRIB3 knockdown by western blotting. Cells transduced with SCR or shTRIB3 subjected to HG stimulation; (B) Levels of BAX, BCL-2, and TRIB3 expression examined via western blotting assay; (C) Cell viability assessed by CCK-8; (D) Flow cytometry analysis determines the apoptosis level; (E) Expression of TNF-α, IL-6, and IL-1β measured by ELISA. TRIB3: Tribbles pseudokinase 3; HG: High glucose; SCR: Scrambled (control group in experiments involving RNA interference); shTRIB3: Short hairpin RNA targeting TRIB3; BAX: Bcl-2-associated X protein; BCL-2: B-cell lymphoma 2; CCK-8: Cell Counting Kit-8; TNF-α: Tumor necrosis factor alpha; IL-6: Interleukin 6; IL-1β: Interleukin 1 beta; ELISA: Enzyme-linked immunosorbent assay; GAPDH: Glyceraldehyde 3-phosphate dehydrogenase.

Considering that TRIB3 expression is upregulated in renal tissue of diabetic mice and podocytes by HG stimulation, we sought to explore how inhibiting TRIB3 could alleviate podocyte injury induced by HG. shRNA targeting TRIB3 effectively knocked down endogenous TRIB3 expression in MPC5 cells (Figure 3A), leading to a marked decrease in TRIB3 levels both with and without HG stimulation (Figure 3B). The viability of MPC5 cells, which was significantly impaired by HG treatment, was notably improved upon TRIB3 knockdown (Figure 3C). Flow cytometry analysis showed that apoptosis in MPC5 cells induced by HG was mitigated following TRIB3 silencing (Figure 3D). This apoptotic reduction was evidenced by the upregulation of the anti-apoptotic protein B-cell lymphoma 2 (BCL-2) and the downregulation of the pro-apoptotic protein BAX (Figure 3B). Subsequent analyses revealed elevated levels of pro-inflammatory cytokines TNF-α, IL-6, and IL-1β in supernatants of HG-stimulated MPC5 cells, indicating an inflammatory response (Figure 3E). However, TRIB3 knockdown reversed these effects, thereby implicating TRIB3 in modulating inflammatory processes that contribute to the pathogenesis of DN, which is characterized by kidney inflammation, podocyte, and endothelial cell injury [32, 33].

### Inhibition of TRIB3 blocked HG-induced extracellular matrix (ECM)-related protein expression in MPC5 cells

**Figure 4.**
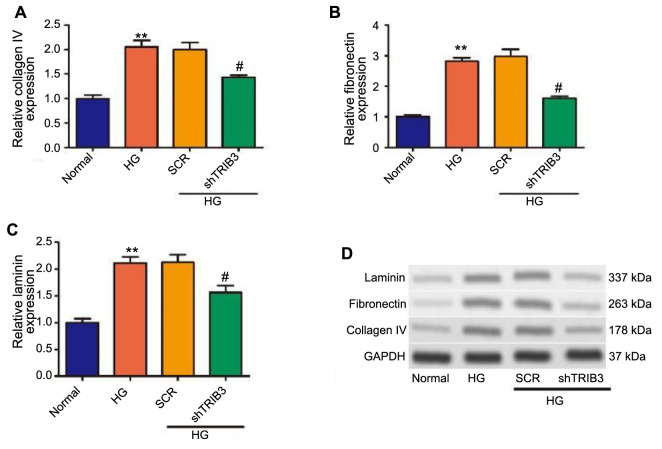
**Effects of TRIB3 silencing on the expression of ECMs in MPC5 cells.** **P < 0.01 vs normal group; ^#^*P* < 0.01 vs the SCR group. (A–C) Effects of decreasing TRIB3 expression on the mRNA level of collagen IV, fibronectin, and laminin by qRT-PCR; (D) The protein of collagen IV, fibronectin, and laminin detected by western blotting assay. TRIB3: Tribbles pseudokinase 3; ECM: Extracellular matrix; SCR: Scrambled (control group in experiments involving RNA interference); qRT-PCR: Quantitative reverse transcription polymerase chain reaction; GAPDH: Glyceraldehyde 3-phosphate dehydrogenase.

Accumulation of ECM is a hallmark feature of DN and contributes to the structural and functional changes in the kidneys [34]. Collagen IV, fibronectin, and laminin were reported to be markedly high in the kidneys of mice with diabetic kidney disease [35]. As shown in Figure 4A–4C, qRT-PCR revealed that the mRNA expression of ECM-related genes (collagen IV, fibronectin, and laminin) was markedly high in HG-induced MPC5 cells, which could be inhibited by TRIB3 knockdown. Additionally, TRIB3 silencing decreased the expression of ECM components, including collagen IV, fibronectin, and laminin in MPC5 cells treated with HG (Figure 4D). These results preliminarily demonstrated that TRIB3 played an important role in the process of renal fibrosis.

**Figure 5.**
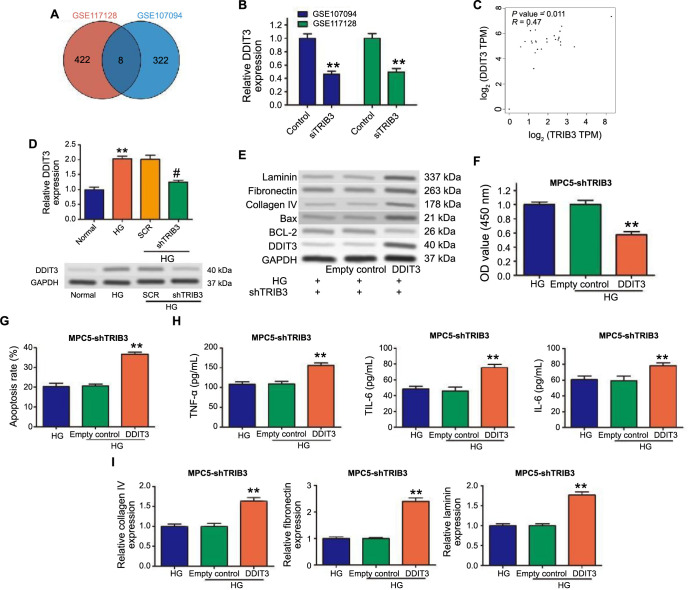
**DDIT3 involved in TRIB3-regulated inflammatory response and ECM accumulation.** **P < 0.01 vs normal group; ^#^*P* < 0.01 vs the SCR group. (A) Venn diagram of DEGs from the GSE117128 and GSE107094; (B) DDIT3 expression in GSE117128 and GSE107094; (C) Spearman correlation coefficient analysis used to analyze the association of TRIB3 and DDIT3 in cortex of kidney; (D) mRNA and protein of DDIT3 detected by qRT-PCR and western blotting, respectively; (E) Western blot analysis showed the expression of DDIT3, BCL-2, BAX, collagen IV, fibronectin and laminin under indicated treatment; (F) Cell viability assessed through CCK8 assay; (G) Flow cytometry illustrated the apoptosis rate of MPC5 cells; (H) Levels of inflammatory cytokines in cell supernatant assessed by ELISA; (I) mRNA levels of collagen IV, fibronectin, and laminin measured by qRT-PCR. DDIT3: DNA-damage-inducible transcript 3; TRIB3: Tribbles pseudokinase 3; ECM: Extracellular matrix; SCR: Scrambled (control group in experiments involving RNA interference); DEGs: Differentially expressed genes; qRT-PCR: Quantitative reverse transcription polymerase chain reaction; BCL-2: B-cell lymphoma 2; BAX: Bcl-2-associated X protein; CCK8: Cell Counting Kit-8; ELISA: Enzyme-linked immunosorbent assay; GAPDH: Glyceraldehyde 3-phosphate dehydrogenase.

**Figure 6.**
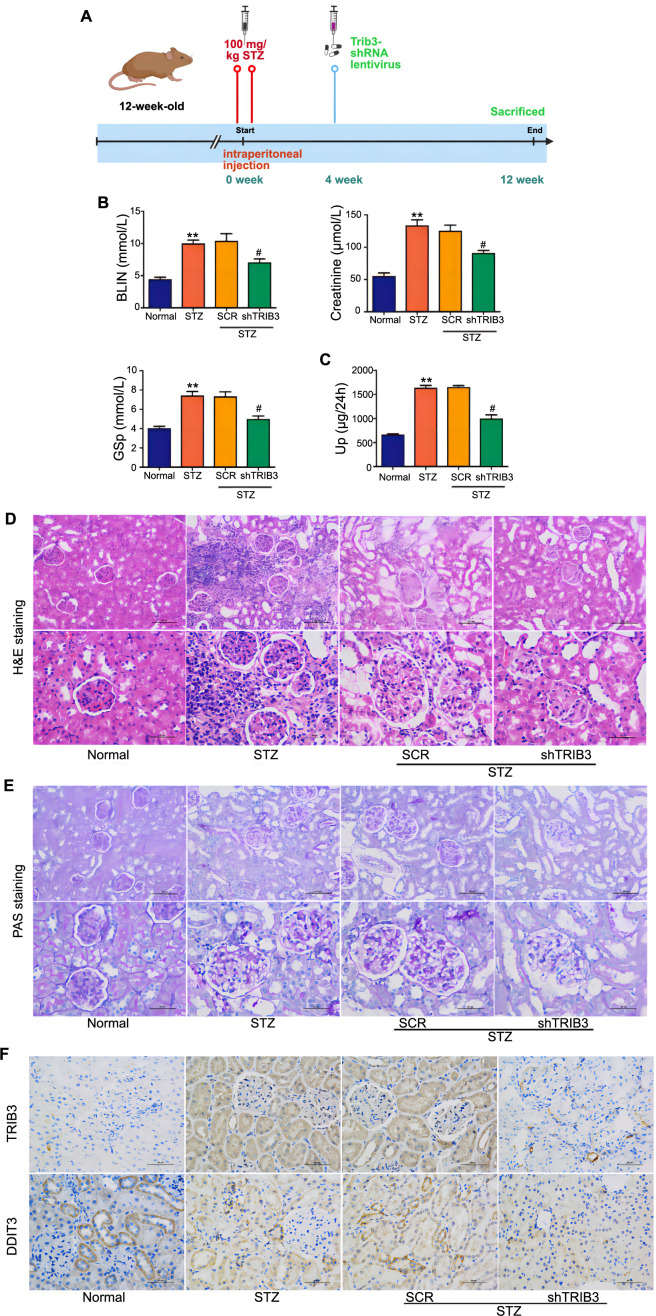
**TRIB3 silencing improved renal function in vivo.** **P < 0.01 vs normal group; ^#^*P* < 0.05 vs SCR group. (A) The level of creatinine, BUN, and GSP in serum measured to assess glucose metabolism and kidney damage; (B) UP levels in urine measured to assess kidney damage; (C) Renal tubular histopathological analysis in the kidneys of the mice detected by H&E (Magnification: 200 and 400×); (D) PAS staining of kidney tissues from mice; (E) Immunohistochemical staining of TRIB3 and DDIT3 in kidney sections (Magnification: 400 ×). TRIB3: Tribbles pseudokinase 3; SCR: Scrambled (control group in experiments involving RNA interference); BUN: Blood urea nitrogen; GSP: Glycated serum protein; UP: Urinary protein; H&E: Hematoxylin and eosin; PAS: Periodic acid-Schiff; DDIT3: DNA-damage-inducible transcript 3.

### TRIB3 promoted podocyte cytoskeleton via upregulating DDIT3

To further explore the underlying molecular basis of TRIB3 in the development of DN, GSE117128 and GSE107094 were used to screen DEGs after TRIB3 knockdown according to the threshold of |log2 FC | > 1 and adjusted *P* value < 0.05. Six common downregulated genes, including *GPC4, CCDC18-AS1, DDIT3, TFPI, ZC3H6,* and *ENPP1*, were identified after TRIB3 knockdown by Venn diagram (Figure 5A). DDIT3 was selected for further investigation due to its involvement in exacerbating fibrotic tissue remodeling [21, 36]. The levels of DDIT3 were significantly reduced in GSE117128 and GSE107094 datasheets (Figure 5B). A positive relationship was observed between TRIB3 and DDIT3 in the cortex of the kidney based on the online Gene Expression Profiling Interactive Analysis (GEPIA2) (Figure 5C). DDIT3 expression was also observed to be significantly upregulated in HG-induced MPC5 cells, which could be inhibited by TRIB3 knockdown both at mRNA and protein levels (Figure 5D).

To evaluate the roles of DDIT3 in the biological effects of TRIB3 on HG-induced MPC5, DDIT3 was overexpressed in TRIB3 knockdown in MPC5 cells under the HG environment (Figure 5E). Functional analysis experiment results showed that DDIT3 overexpression suppressed cell viability (Figure 5F) and promoted cell apoptosis (Figure 5G). Furthermore, DDIT3 overexpression partly abolished the decreased level of TNFα, IL-6, and IL-1β repressed by TRIB3 knockdown (Figure 5H). We observed that the downregulation of collagen IV, fibronectin, and laminin was effectively abrogated when DDIT3 was overexpressed (Figure 5I). In addition, ectopic DDIT3 expression led to the opposite effects following TRIB3 silencing under the HG state (Figure 5B). Our findings indicated that TRIB3 promoted the progress of DN mainly through increasing the expression of DDIT3.

### Inhibition of TRIB3 prevented renal interstitial fibrosis in vivo

To further assess the role of TRIB3 in renal interstitial fibrosis in DN, TRIB3-shRNA lentiviruses were injected into the kidneys of STZ-induced diabetic mice via the tail vein. The serum creatinine, BUN, and glucose metabolism GSP levels of diabetic mice were significantly higher than in healthy mice (Figure 6A), whereas those in shTRIB3 groups with DN were significantly decreased compared with those in the model group (Figure 6A). A similar result was observed in 24 h UP (Figure 6B). Furthermore, H&E staining results showed that the glomerular injury and infiltration of inflammatory cells increased in the kidney tissues of diabetic mice compared with the normal group, while these changes were notably reversed after TRIB3 silencing (Figure 6C). PAS staining showed that more severe glomerular mesangial expansion was observed in diabetic mice, whereas these changes were attenuated with TRIB3 silencing (Figure 6D). IHC staining showed that the intensity of TRIB3 and DDIT3 was increased in diabetic mice, while the expression of TRIB3 and DDIT3 was decreased after TRIB3-shRNA lentiviruses treatment (Figure 6E). These results indicated that TRIB3 promoted the progression of kidney injury in diabetic mice via the upregulation of DDIT3.

**Figure 7.**
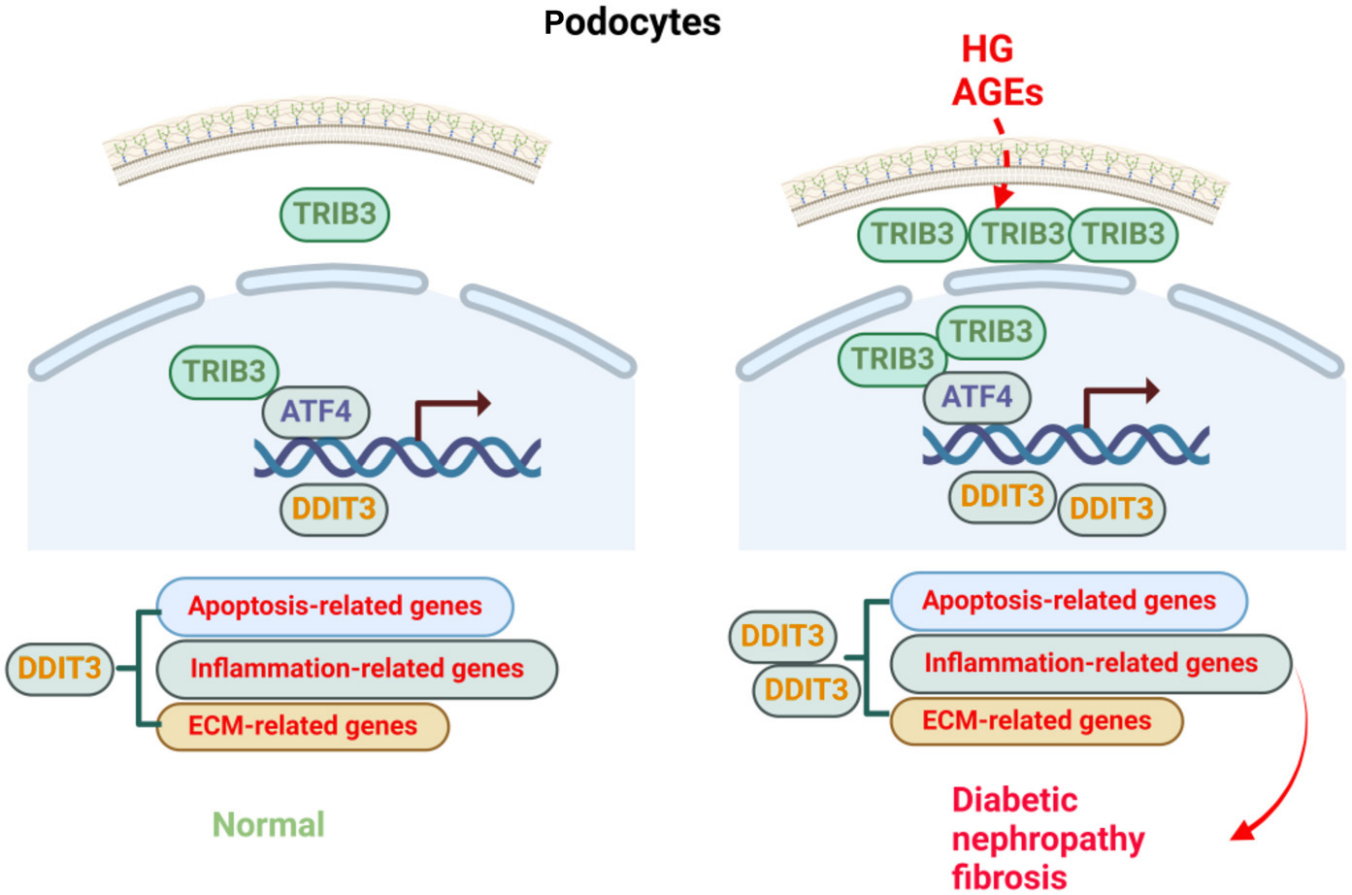
**Schematic diagram illustrating the proposed molecular mechanism TRIB3-DDIT3 axis in DN.** Under hyperglycemic conditions, TRIB3 was overexpressed and led to upregulation of DDIT3. ATF4 was reported to interact with TRIB3 and mediated DDIT3 expression. TRIB3 promoted the progress of DN by upregulating the expression of DDIT3 at transcriptional activation under HG conditions. TRIB3: Tribbles pseudokinase 3; DDIT3: DNA-damage-inducible transcript 3; DN: Diabetic nephropathy; ATF4: Activating transcription factor 4; HG: High glucose; AGEs: Advanced glycation end products.

## Discussion

DN is a serious complication of diabetes that affects a large number of people worldwide, and its incidence and prevalence continue to rise [26, 37]. Previous studies have shown that TRIB3 was significantly increased in DN and inhibition of TRIB3 expression could reduce glomerular damage and albuminuria in diabetic animal models [9, 38]. In the current article, GEO database analysis showed that in contrast to normal podocytes, TRIB3 expression was remarkably higher in HG-induced podocytes and renal cortical tissues from STZ-induced diabetes. Our data confirmed that TRB3 expression levels were upregulated in HG-induced MPC5 cells and renal tissues from diabetic mice both at mRNA and protein levels. Furthermore, shRNA-mediated TRIB3 knockdown was used to decrease endogenous TRIB3 expression, and TRB3 silencing abolished HG-induced apoptosis, inflammatory response, and ECM accumulation in MPC5 cells by reducing the expression of DDIT3, a crucial gene in regulating cellular adaptation and survival during stressful conditions. In vivo experiments verified that TRB3 silencing ameliorated nephropathic symptoms in diabetic mice.

Numerous studies have shown that TRB3 mediates diverse and intricate cellular processes by interacting with transcription factors or proteins to act as mediators of cellular stress responses [9, 17, 39]. TRIB3 was considered a nutrient sensor and influenced energy metabolism by regulating intracellular signaling pathways, including AKT signaling cascade, MAPK pathways, and signaling pathways associated with β-cell apoptosis under heightened nutrient influx, insulin resistance, and elevated blood glucose levels [40–42]. TRIB3 has reported to act as a pivotal regulator of crucial cellular processes through its interactions with transcription factors such as CCAAT-enhancer-binding protein homologous protein (CHOP), peroxisome proliferator-activated receptor alpha (PPARα), and activating transcription factor 4 (ATF4), including glucose and lipid metabolism, adipocyte differentiation, autophagy, proteasomal degradation, and apoptosis [12, 39, 43]. A recent report showed that compared to healthy subjects, TRIB3 level was significantly increased in the plasma of diabetic patients, and high TRIB3 level was associated with fasting blood glucose and insulin resistance [44]. The activation of TRIB3 in hyperglycemic conditions can be attributed to a complex interplay of oxidative stress, endoplasmic reticulum (ER) stress, inflammatory signaling, and disruptions in nutrient sensing. Each of these pathways contributes to the upregulation of TRIB3, which in turn plays a pivotal role in managing the cellular responses to hyperglycemia, particularly in the context of DN. In our present study, short hairpin RNA-mediated TRIB3 knockdown attenuated the inhibition of cell viability of HG-induced MPC5 cells. Furthermore, TRIB3 silencing decreased cell apoptosis, proinflammatory cytokine secretion, and ECM-related protein expression in MPC5 cells in the presence of high glucose environments, consistent with previous reports.

To investigate the potential mechanisms, DEGs from the GEO database were analyzed and DDIT3 was identified as a gene of interest based on its biological functions. DDIT3 is a downstream target of the unfolded protein response (UPR) pathway activated during ER stress, which is a hallmark of DN [21]. When ER stress is prolonged or severe, DDIT3 expression is upregulated, and it promotes apoptosis by regulating the expression of pro-apoptotic genes [45]. In fibrotic conditions, persistent ER stress can lead to excessive cell death, contributing to tissue damage and fibrosis [21, 46, 47]. DDIT3 has been shown to regulate the expression of TRIB3 under conditions of ER stress. This interaction has been observed in studies investigating cellular responses to different stressors, including glucose deprivation and hypoxia [46, 48]. In our study, we found that DDIT3 was significantly decreased after TRIB3 knockdown both at mRNA and protein levels. Further functional studies clearly showed that overexpression of exogenous DDIT3 effectively abolished the inhibition effect of TRIB3 silencing on cell apoptosis, proinflammatory cytokine secretion, and ECM-related protein expression, indicating that TRIB3 contributed to progress of DN in a DDIT3-dependent manner. In addition, the observed reduction in glomerular damage and albuminuria through the inhibition of TRIB3 in diabetic animal models underscores its critical involvement in the pathogenesis of DN.

## Conclusion

Our study shows that targeting TRIB3 holds great therapeutic value for DN and inhibition of TRIB3 significantly attenuates podocyte injury induced by HG. Meanwhile, our findings unveiled a novel mechanism wherein TRIB3 upregulated the expression of DDIT3 (Figure 7), which was associated with excessive or prolonged activation under pathological conditions.

## Data Availability

All of the data presented in this study are available upon request to the corresponding authors.
